# Ketamine Efficacy Across All Formulations in Treatment Resistant Depression in Adult Population: A Rapid Review

**DOI:** 10.1192/bjo.2024.173

**Published:** 2024-08-01

**Authors:** Bikramaditya Jaiswal, Neha Singh, Benson Benjamin

**Affiliations:** 1 East London NHS Foundation Trust, London, United Kingdom.; 2 North East London NHS Foundation Trust, London, United Kingdom

## Abstract

**Aims:**

The primary aim of this rapid review was to evaluate the evidence base for the efficacy of ketamine across all formulations and routes of administrations in the treatment of adult patients with treatment resistant depression (TRD).

**Methods:**

This rapid review retrieved controlled trials on use of ketamine across all of its formulations, including all isomers and across all routes of administration in TRD patients for achieving response and remission. This review included PubMed and PsycINFO databases. The retrieved studies were screened with the help of a screening tool and data were extracted by using data extraction forms by two authors. The studies were evaluated for quality of evidence, ethical issues and critically analyzed. Narrative synthesis was used for data synthesis.

**Results:**

This review retrieved 10 placebo controlled randomized controlled trials (RCT) on intravenous (IV) ketamine, IV esketamine, intranasal (IN) ketamine and IN esketamine in TRD patients. IV ketamine and esketamine showed higher rates of remission and response in comparison with placebo groups in TRD patients. There was no significant improvement in response and remission rates in TRD patients on IN esketamine in comparison with placebo. The adverse effects in the intervention groups were of mild to moderate severity and short lasting mostly resolving within a day.

**Conclusion:**

This review recommends IV ketamine and esketamine can help in achieving early response and remission in TRD patients and it seems to be a well-tolerated treatment option. Further studies are needed to assess these issues around safety, ease of administration and potential for dependence.

